# Retraction Note: Rapid Onset Gender Dysphoria: Parent Reports on 1655 Possible Cases

**DOI:** 10.1007/s10508-023-02635-1

**Published:** 2023-06-14

**Authors:** Suzanna Diaz, J. Michael Bailey

**Affiliations:** 1 Parents of ROGD Kids, New York, NY USA; 2 Department of Psychology, Northwestern University, 2029 Sheridan Rd., Evanston, IL 60208 USA

**Retraction Note: Archives of Sexual Behavior (2023) 52:1031–1043** 10.1007/s10508-023-02576-9

The Publisher and the Editor-in-Chief have retracted this article due to noncompliance with our editorial policies around consent. The participants of the survey have not provided written informed consent to participate in scholarly research or to have their responses published in a peer reviewed article. Additionally, they have not provided consent to publish to have their data included in this article. Table 1 and the Supplementary material have therefore been removed to protect the participants’ privacy.

The authors disagree with this retraction.

